# Deterioration of direct restorative materials under erosive conditions with impact of abrasion and attrition *in vitro*

**DOI:** 10.1080/26415275.2023.2202211

**Published:** 2023-06-09

**Authors:** Aida Mulic, Amund Ruud, Ida R. Stenhagen, Ellen Bruzell, Amela Tulek

**Affiliations:** Nordic Institute of Dental Materials (NIOM), Oslo, Norway

**Keywords:** Surface roughness, hardness, substance loss, profilometry

## Abstract

**Objective:**

To compare the cumulative impact of sequential wear on mechanical properties and appearance of a composite resin (CR), Filtek Z250^®^, a glass ionomer GI, Fuji IX GP^®^, and a glass hybrid (GH), Equia Forte^®^.

**Material and Methods:**

Six equally sized specimens of each material were subjected to wear tests, i.e., simulation of brushing, chewing and acidic liquid exposure, mimicking at least 6 months of clinical exposure. Surface roughness, hardness, substance loss and degree of shade lightness were determined.

**Results:**

Following wear tests, significant increase in surface roughness and decrease in hardness values were observed for all materials (*p* < .05). Significantly larger substance loss was found in Equia Forte^®^ specimens compared to Filtek Z250^®^ (*p* < .05), while that of Fuji IX^®^ exceeded the measurement capacity of the instrument. Opposite to the two other materials, the shade of Filtek Z250^®^ became darker.

**Conclusions:**

Sequential wear exposure mimicking abrasion, erosion and attrition to products representing CR, GI and GH, caused weakening and change in appearance of the materials. The composite resin was the most mechanically resistant to the sequential wear.

## Introduction

Tooth wear is an irreversible, physiological phenomenon that may be classified into several categories: abrasion (tooth wear in the presence of a foreign medium), erosion (acid-induced tooth wear) and attrition (wear as a consequence of tooth-to-tooth contact) [1]. Excessive consumption of acidic foods or regurgitation of the gastric acid in the oral cavity may chemically induce tooth substance loss [2,3]. Moreover, when acid softens the tooth surface, it becomes susceptible to physical impacts, namely abrasion and attrition [4]. The process of wear is more severe in some patients, for example those suffering from parafunctional habits [5]. Excessive wear often causes exposure of dentine canals and tooth pulp. Clinically, this manifests as hypersensitivity and pain, reduced chewing efficiency and discolorations [6,7].

Together with the physical wear of abrasion and attrition, erosion can have an impact on the longevity of a restoration [8]. Erosive damage is less severe towards restorative dental materials than dental enamel [9]; however, over time it may cause degradation of certain restorative material components. Replacing a failed restoration comprises more than 50% of all operative procedures. It represent a cost to the patient and contributes to the ‘restoration cycle’, causing dental substance to be removed each time [10]. Composite resins (CR) glass ionomer (GI) and glass hybrid (GH) are the materials most often used for direct restorations, with CR generally being the material of choice [11]. CRs are used in both anterior and posterior dentition due to their favorable optical, mechanical or biological characteristics over other direct restorative materials [12]. GIs are used in restorative dentistry due to their high degree of biocompatibility, adhesion to tooth structure, fluoride release and easy handling [13–15]. Their main indications are class V and root surface restorations, primary dentition and atraumatic restorative treatment for caries lesions. However, one of the clinical limitations is rough surface texture, which can hamper mechanical resistance and contribute to plaque formation [16]. Newer generations of high-viscosity GH materials, such as Eqiua Forte^®^, are claimed to have improved physical and mechanical properties compared to earlier GIs and extended indications to non-bearing and load-bearing class I and II restorations [17,18]. GIs are generally not recommended as solely restorative materials in the load-bearing areas due to their physical and mechanical properties [19]. The suitability of such material for posterior regions is currently? debated [17,20,21]. Conflicting results regarding the indications and preferred material generate the need for further evaluation of bulk-filled glass hybrid materials versus conventional restorative materials [22].

The aim of this study was to determine and compare the level of surface roughness, hardness, substance loss, and change in degree of shade lightness following sequential exposure to abrasion, erosion and attrition in three dental materials, each representing three direct restorative material categories; composite resins, glass ionomers and glass hybrids.

## Material and methods

The investigated products were the CR, Filtek Z250^®^, the GI, Fuji IX GP^®^, and the GH, Equia Forte^®^, with the resin, Equia Forte Coat^®^ (Table 1).

**Table 1.** Specifications of the restorative materials subjected to wear tests according to manufacturers. CR: composite resin; GIC: glass ionomer cement; GH: glass hybrid.

Materials	Composition	Type	Shade	Indications	Manufacturer
Filtek Z250®	Bisphenol A diglycidyl ether dimethacrylate, urethane dimethacrylate, and bisphenol A polyethylene glycol diether dimethacrylate, filled with 60% (volume) silica/zirconia	Microhybrid CR	A2	Direct anterior and posterior restorations, core buildup, splinting, indirect restorations including inlays, onlays and veneers.	3M ESPE, St Paul, MN, USA
Fuji IX®	Fluoroaminosilicate glass; polyacrylic acid; polybasic carboxylic acid; water	Conventional GI	A2	Geriatric and pediatric restorations, final restorations (non-stress areas), intermediate restorations, core build-up and long-term, temporary restorations.	CG Corporation, Tokyo, Japan
Equia Forte®	Strontium fluoroaluminosilicate glass; polyacrylic acid; aqueous polyacrylic acid; water	GH	A2	Class I, II and V restorations, intermediate restorations, core build-up material.	CG Corporation, Tokyo, Japan
Equia Forte Coat®	Methyl methacrylate; camphorquinone.				

### Preparation of specimens

**Figure 1.**
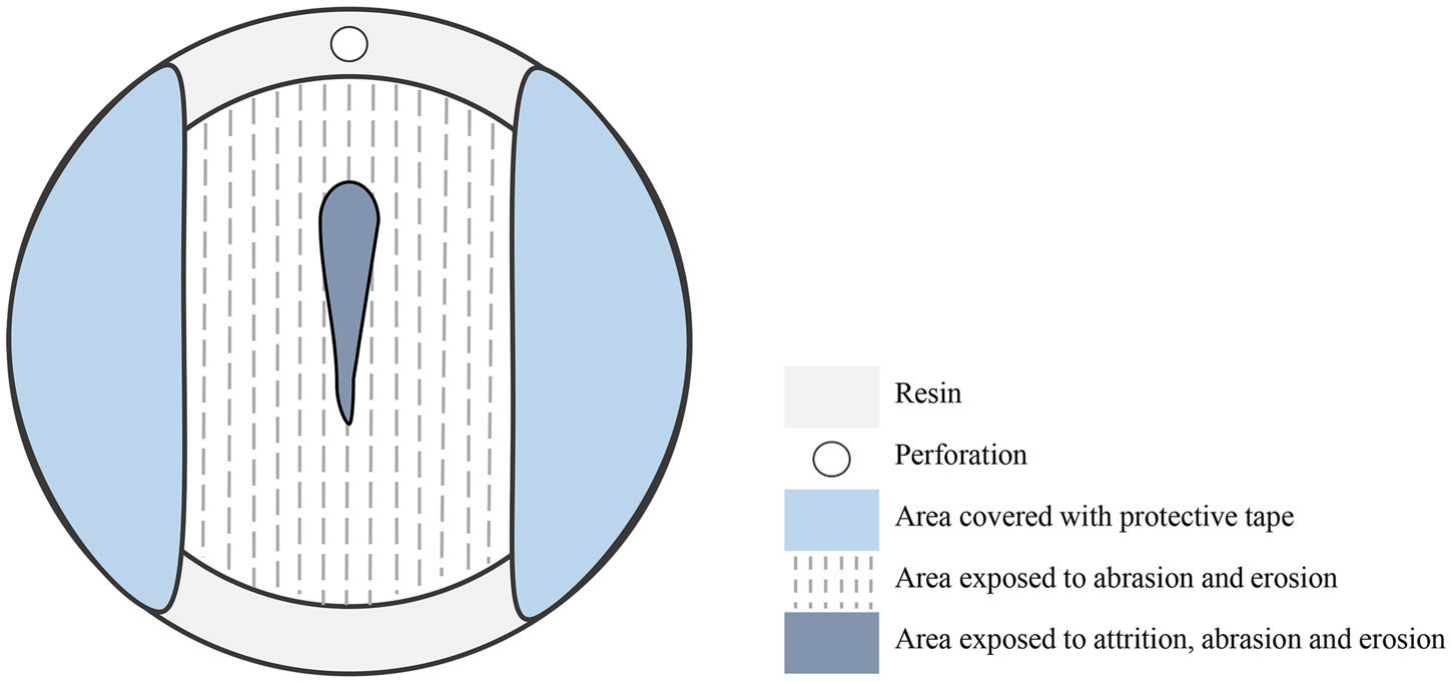
Schematic illustration of the different zones of a resin specimen being subjected to abrasion, erosion and attrition.

One operator (AT) prepared all specimens according to the manufacturers’ instructions at room temperature (21 °C ± 1 °C) (Figure 1). A cylindrical stainless-steel mold (inner Ø 20 mm, depth 2 mm) was filled with each material, isolated with cellulose strips and firmly pressed between two metal plates in a manual press for 10 s to squeeze out excess material. The specimens were light-cured (Demi Ultra, Kerr, Brea, CA, USA; light tip Ø: 0.807 cm; irradiance: 1236 mW/cm^2^ ± 5.4%, λ_max_= 469 nm) as measured with spectrometer and integrating sphere as described in [23] for 20 s at the center of the specimen and at the eight following positions, covering the outer part of the specimen: 12, 1, 3, 5, 6, 8, 9, and 11 O’clock. Finally, Equia Forte^®^ specimens were covered with Equia Forte Coat^®^ (CG Corporation, Tokyo, Japan). According to the manufacturer’s instructions, the coat was applied with a microbrush, and light-cured for 20 s.

### Embedding, grinding, and polishing

Six specimens of each material group were placed in a round teflon mold (Ø 25 mm) with the test surface facing the bottom of the mold. A two-component clear acrylic mounting system (ClaroCit, Struers ApS, Ballerup, Denmark) was prepared by hand mixing the powder and liquid for 60 s. The mixture was poured into the mold covering the entire surface of the specimen, with a thickness of 3 mm. Curing time was approximately 30 min at controlled room temperature (21 °C ± 1 °C).

In order to standardize the polishing procedure, the specimens were plane-grinded and polished in a semi-automatic polishing machine. The specimens were mounted in a purpose- built specimen holder attached to the rotor of the polishing machine. Grinding was performed under constant water cooling, using US # 320-, 500-, 1200-grit silicon carbide grinding papers, at 150 rpm for 30 s. The specimens were polished on a composite-covered plate under constant lubrication (agent containing 9 µm diamond particles) at 300 rpm for another 30 s. All grinding/polishing equipment was provided by Struers ApS, Ballerup, Denmark. After each grinding/polishing step, specimens were thoroughly rinsed under running tap water for 20 s to remove any residual particles from the grinding procedure and finally air-dried. Prior to the experimental procedure, adhesive tape (Scotch 389 PE Coated White Duct Tape, 3 M Company, Neuss, Germany) was placed on the specimens to ensure protected portions of the surface from the erosive and abrasive exposure tests as shown in Figure 1. When not used in the experimental procedure or analyzed, specimens were stored in a desiccator at 37 °C in artificial saliva prepared in-house (0.7 mmol/l CaCl_2_, 0.2 mmol/l MgCl_2_, 4.0 mmol/l KH_2_PO_4_, 30.0 mmol/l KCl, 20.0 mmol/l Hepes, pH 7.0) [24].

### Wear tests

#### Abrasion

Two specimens were firstly fixed in two plastic chambers of an in-house designed and custom-built brushing machine. Two toothbrushes with soft, flat bristles (Oral B, Procter & Gamble, Cincinnati, OH, USA) were fixed in metal holders, one for each chamber. Each brush produced a force of 2 N and a stroke length about 35 mm. The machine was set to 75 000 cycles (150 000 strokes; ∼12 h) equivalent to 2 min of brushing twice a day for 6 months [8]. The chambers were filled with a prepared abrasive slurry according to ISO 11609: 2017 [25]. A new pair of toothbrushes was used for each material. After abrasion, the specimens were carefully removed from the holder, rinsed with double distilled water (ddH_2_O, type 2) and air-dried. The specimens were kept in artificial saliva until the following procedure.

#### Erosion

Following abrasion, specimens were placed in a liquid cycler containing a customized, in-house designed holder and two chambers immersed into a bath filled with ddH_2_O (37 °C) to simulate erosion. One chamber was filled with 300 ml artificial saliva (pH 7) and the other with 300 ml acidic drink (Pepsi MAX^®^, pH 2.3). An electrical motor moved the holder with specimens between the chambers. A plastic cover was placed over the instrument to reduce evaporation. One cycle consisted of soaking the specimens into artificial saliva for 120 s, holding in air for 30 s to drain off the liquid before immersing the specimens in the acidic drink for 120 s, and again holding in air for 30 s (i.e. 1 cycle = 300 s). During the hold period, the specimens were oscillated in air with a short-range motion to shake off any excess liquid. According to Attin and Wegehaupt, 2014 [26], 16 h of acidic exposure to hybrid composite resins did not result in any detectable damage. To provoke an effect, a doubling of exposure time to 32 h (375 cycles) was therefore chosen. This exposure time corresponds to more than 6 months of clinically estimated acid drink intake. The pH values of the acidic drink and artificial saliva in the chambers were monitored (Sension pH31, Hatch, Manchester, UK). The liquids were replaced every 8 h to avoid change in pH. Following cycling, specimens were washed with ddH_2_O and stored in artificial saliva until the next experimental procedure.

#### Attrition

**Figure 2.**
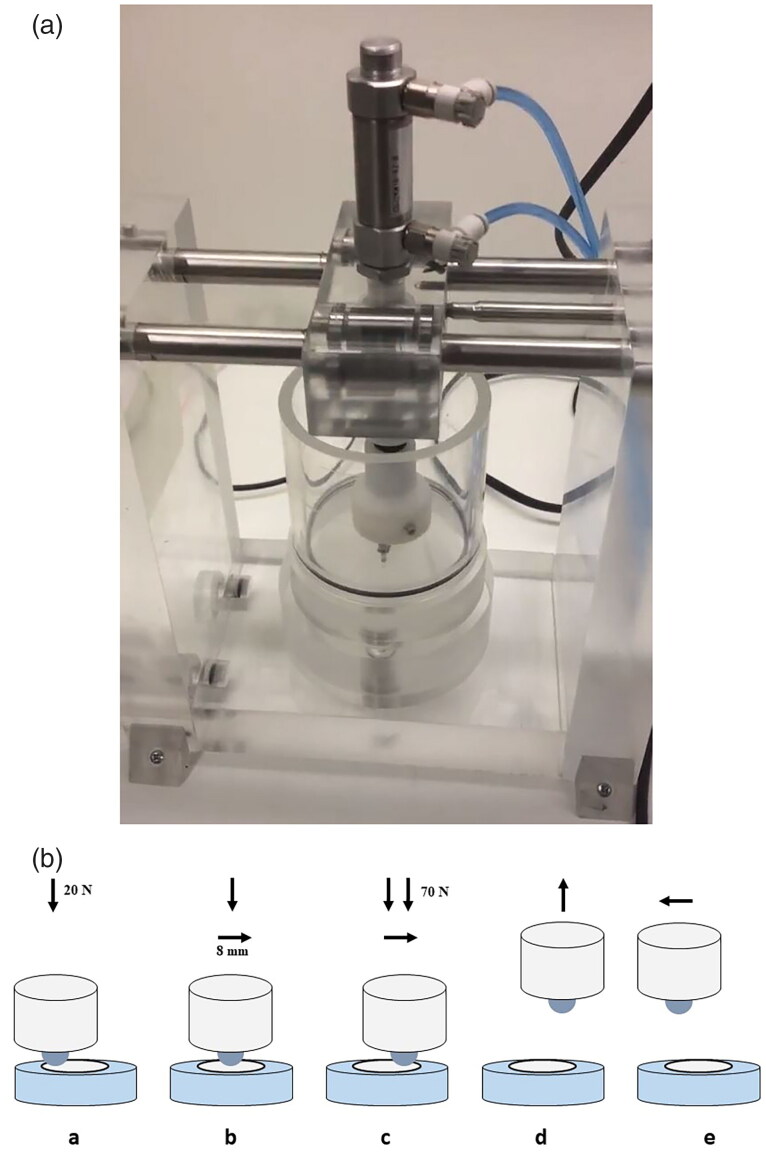
Chewing simulator used in the attrition experiment. (A) Image of the simulator showing chamber and piston with the antagonist holder. (B) Schematic representation of chewing simulation. (**a**) The chewing machine pushes the antagonist (white cylinder; a stainless steel ball (Ø 4 mm) embedded in epoxy) against the specimen (blue cylinder) by applying a force of 20 N downward; (**b**) antagonist moves across the specimen to the opposite side (8 mm) while maintaining the force; (**c**) the force increases to 70 N before the end of the slide movement, and the movement continues until the stroke is finished; (**d**) after the antagonist reaches the end of the stroke, it moves upwards; (**e**) the antagonist moves to the starting position to finish one cycle.

The final wear test was conducted in a customized, in-house designed chewing simulator with a single chamber filled with 20 ml of artificial saliva (pH 7) and a specimen holder on the bottom (Figure 2(A,B)). A stainless steel ball (Ø 4 mm) acting as an artificial antagonist was embedded in epoxy (ClaroCit, Struers ApS) and mounted in the upper, opposing holder of the chewing simulator. The antagonist was adjusted to be in contact with the specimen transferring a force of 20 N and slid horizontally across its surface for a distance of 8 mm, producing attrition wear. At the end of the chewing motion, an additional static load of 70 N was applied to produce localized wear (Figure 2(B)). Each specimen was subjected to 125 000 cycles with cycle time of 1 s. This procedure is equivalent to 6 months of human mouth chewing [27].

### Measurement procedures

Surface roughness, hardness and shade lightness determination were performed prior to and after the wear procedures, while profilometric analyses of the substance loss were performed only after the wear procedure.

#### Surface roughness and hardness

Roughness of the surface, Ra (µm), was measured using the surface profilometer (Mitutoyo SJ 201 P, Mitutoyo Scandinavia AB, Upplands Väsby, Sweden) in five different positions on the specimen surface. The surfaces of the specimens were visually inspected.

Vickers hardness (HV0.5) was measured with Innovatest Nova 300 hardness testing machine (Duramin 40 A1, Struers) using a loading mass of 500 g and a dwell time of 15 s. A Vickers diamond-shaped indenter with a square base was pressed vertically onto the specimen surface in five different positions.

#### Shade determination

The shade of the specimens was determined independently by two calibrated clinicians (A.M. and A.T.) (*n* = 2; mean inter-examiner agreement (κw)=0.55) using a visual detection method (VITA classical A1-D4^®^ shade guide, VITA Zahnfabrik, Bad Säckingen, Germany). In addition, both clinicians measured the shade using a digital spectrophotometer (VITA Easyshade^®^ V; VITA Zahnfabrik). The shades of Vita Classical is divided into four categories designated by the letters A, B, C, and D corresponding to reddish-brownish, reddish-yellowish, grayish, and reddish-gray, respectively. In the present study, the shades were sorted in steps in order of highest to lowest degree of lightness: B1; A1; B2; D2; A2; C1; C2; D4; A3; D3; B3; A3.5; B4; C3; A4; C4. Numbers from 1 to 16 in increasing order were assigned to the shades: B1 = 1, A1 = 2, etc., up to C4 = 16. The shade determinations were performed in a calibrated light cabinet (VeriVide, Cromocol Scandinavia AB, Borås, Sweden equipped with D65 florescent tubes; illuminance range: 1050–1930 lx). The instrument measurements were performed by holding the instrument probe tip at 90° to the surface in the middle of the specimen. The visual and digital evaluations were repeated twice per sample, and the final shade of each specimen was determined by each evaluator and method. One final shade for six specimens of each material group at baseline and postwear exposure was determined by calculating the arithmetic mean of the assigned numbers representing the corresponding shade. If the mean corresponded to a shade that was not representative for the actual specimens, the shade representing the closest numerical value was chosen, for example, 10 (corresponding to D3) was determined to be A3. When two shades were representative for a set of specimens, the closest shade corresponding to the mean numerical value was chosen, for example, a mean of 6.2 was determined to be C1. The final shade determined by the visual and digital method was reported separately.

#### Substance loss

**Figure 3.**
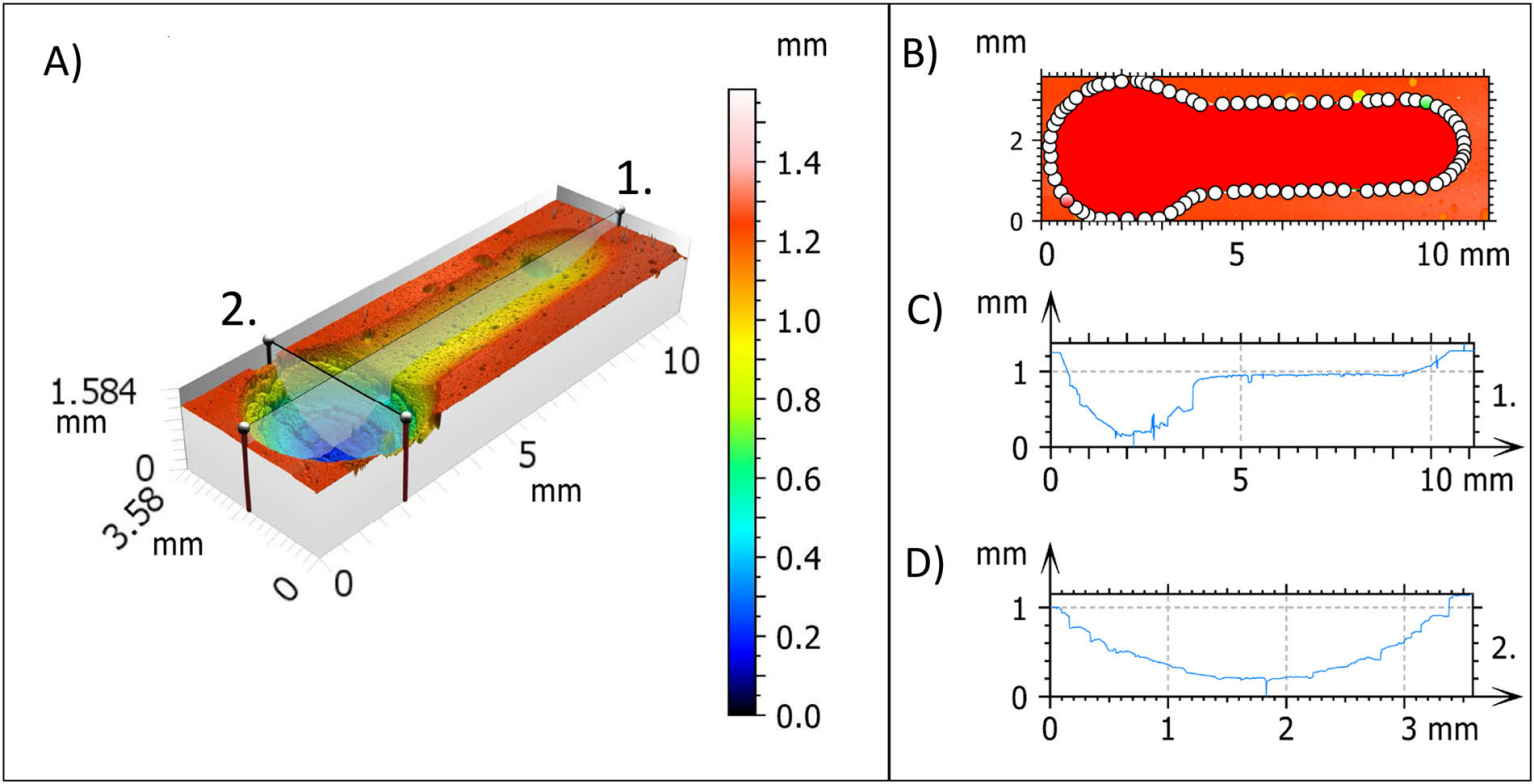
Postexposure profilometry of the worn area (groove). (**A**) 3D image of the worn area of a typical Filtek Z250® specimen. ‘1’ and ‘2’ represent the longitudinal and transversal section, respectively, through the deepest point; (**B**) surface of the worn area; (**C**) longitudinal 2D profile; (**D**) transversal 2D profile.

Repeated sliding of the antagonist over the specimen surface created a groove-shaped worn area. Since the specimen’s surface was previously exposed to abrasion and erosion, this worn area represented the cumulative impact of the three wear methods (Figure 1). Substance loss of the groove was measured with a profilometer using an EPI 20 × v35 objective in the focus variation mode (S Neox optical profiler, Sensofar Measurement System, Barcelona, Spain). Surface, volume and mean depth of the groove were measured (Figure 3). The complete worn region was imaged by a series of 68 micrographs (17 × 4) and stitched with a 10% overlap, analyzed in the SensoSCAN 6.3 software. The dimensions of the scanned area were 13.51 × 2.44 mm^2^ (10472 px × 1894 px). Image analysis and processing of the worn regions were done using SensoMap Standard 7.3 (Sensofar) and Hitachi map 3D standard V8 software (Hitachi, Ltd, Tokyo, Japan) powered by Mountains^®^ 8 (Digital Surf’s Mountains Technology, Besançon, France).

### Statistical analysis

A sample size of six specimens was considered sufficient based on specimen homogeneity and previous experience. Data were analyzed using Graph Pad Prism 9.3.1. Paired *t*-tests were used to determine the difference in roughness and hardness within material groups pre- and post-treatment. One-way ANOVA and Tukey’s multiple comparisons test were used to compare the change in roughness and hardness between the different materials. Two sample *t*-test was used to compare the substance loss of the groove in Filtek Z250^®^ and Equia Forte^®^ specimens. Statistical significance level was set at *p* ≤ .05.

## Results

### Surface roughness and hardness

Prior to treatment the mean surface roughness values of the materials were significantly different from each other (*p* < .05) (Table 2). Following exposure, a 7-fold increase in mean surface roughness was observed in the Filtek Z250^®^ specimens compared to baseline, while the corresponding increases in Fuji IX^®^ and Equia Forte^®^ were 1.7- and 1.8-fold, respectively (*p* < .05) (Table 2). The final mean value of Filtek Z250^®^ was the lowest (Table 2). Visual inspection revealed material flakes coming off the GI and GH specimens that and no traces of toothbrush bristle. The CR surfaces appeared heterogeneous with visible bristle traces.

**Table 2.** Comparison of surface roughness (Ra) and Vickers hardness (0.5 kgf load; HV0.5) values at baseline and after (final) cumulative wear exposure in specimens of three dental materials, each representing a direct restorative category.

Materials	Filtek Z250® resin-based composite			Fuji IX® glass ionomer			Equia Forte® glass hybrid		
Measurement	Baseline (±SD)	Final (±SD)	Change (±SD)	Baseline (±SD)	Final (±SD)	Change (±SD)	Baseline (±SD)	Final (±SD)	Change (±SD)
Mean roughness Ra (µm)	0.13(±0.01)	0.93 (±0.09)*	0.80 (±0.09)	0.86 (±0.05)	1.48 (±0.09) *	0.63(±0.11)	0.65 (±.08)	1.14 (±0.05)*	0.49 (±0.09)
Mean hardness HV0.5	113.6 (±2.95)	100.7 (±1.00)*	11.70 (±2.84)	63.5 (±2.99)	48.3(±1.06) *	14.90 (±3.11)	72.2 (±1.63)	63.0 (±1.06)*	12.09 (±1.40)
Degree of shade lightness (visual)	A3	A3.5	+3	A3	A2	−4	A3	C1	−3
Degree of shade lightness (digital)	A3	A4	+6	A3	A2	−4	A3	C1	−3

Values are expressed as mean (±SD); *n* = 6. (*) significant difference from baseline, paired t‐test, *p* < .05. Shade change was determined as change in degree of lightness across shade categories (VITA classical A1‐D4^®^ shade guide) (visual); VITA Easyshade^®^ V (digital)). A2, A3, A4: ‘reddish brownish’; C1: ‘grayish’. Negative and positive numerical changes in shade lightness indicate lighter and darker shade steps, respectively

Within each material group, the mean hardness value was reduced (*p* < .05) following wear exposure. Between material groups, the changes were of a similar magnitude (*p* > 0.05). The post-exposure mean value of the Filtek Z250^®^ specimens was higher than both the baseline and post-exposure values of the other two materials (*p* < .05) (Table 2).

### Substance loss

**Figure 4.**
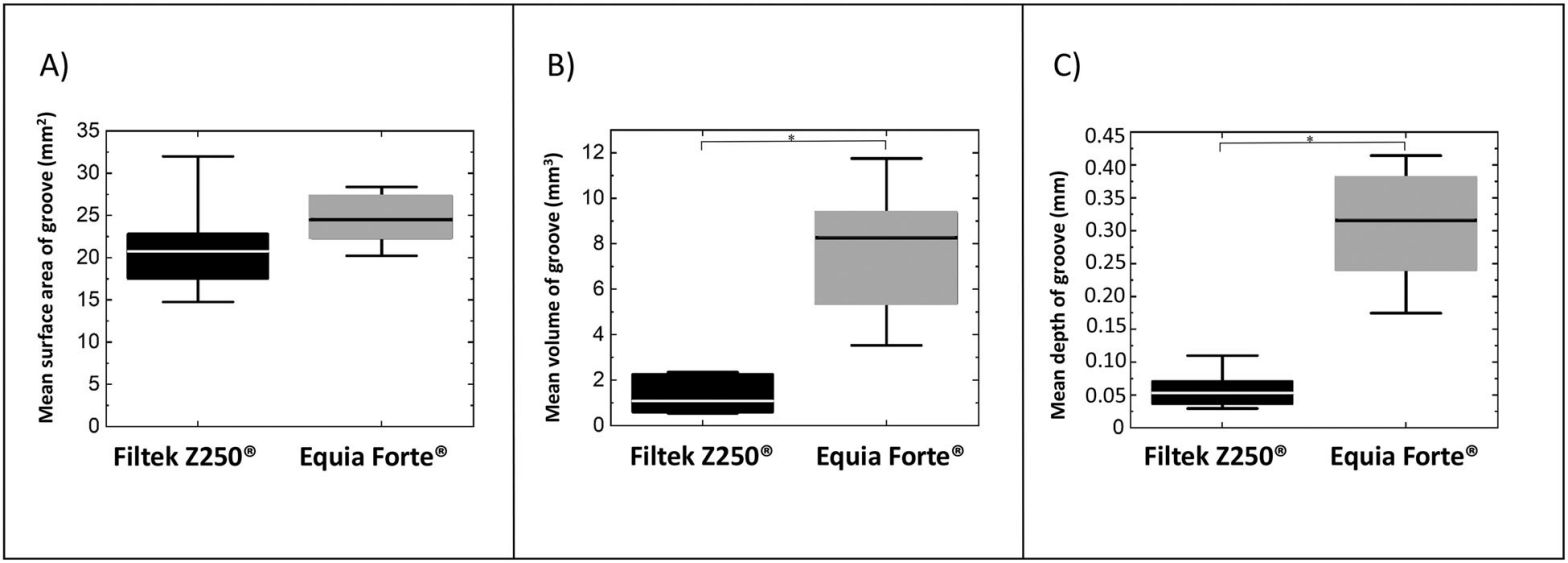
Substance loss of the worn area (groove) for Filtek Z250® and Equia Forte® specimens (*n* = 6) measured by profilometry. Quantities of the worn area: (**A**) surface area (mm^2^); (**B**) volume (mm^3^); (**C**) depth (mm). Box represents 25/75 pecentile; horizontal line in the box represents the mean value; whiskers represent 5-95 percentile; (*) significant difference, *p* <.05. The substance loss of Fuji IX® exceeded the measurement capacity of the optical profilometer.

The mean worn surface areas of Filtek Z250^®^ and Equia Forte^®^ were similar (Figure 4(A)), while the mean volumes (1.31 mm^3^ and 7.38 mm^3^) and mean groove depths (0.06 mm and 0.30 mm) were different for Filtek Z250^®^ and Equia Forte^®^ specimens, respectively (*p* < .05) (Figure 4(B,C)). Profilometry data for Fuji IX GP^®^ could not be obtained as the groove of the specimen was too deep for the optical, profilometer objective to reach the groove surface.

### Shade determination

Following the combined wear exposure, all materials changed degree of shade lightness as determined by both visual and digital methods (Table 2). Only Filtek Z250^®^ became darker. While the Filtek Z250^®^ and Fuji IX^®^ specimens kept their shade category (A; reddish-brownish), the Equia Forte^®^ specimens changed from shade category A to C (grayish). The same final shade lightness was obtained by both visual and digital methods except for the post-wear shades of Filtek Z250^®^ (Table 2).

## Discussion

This *in vitro* study was performed to contribute to selection of a restorative material type by investigating certain dental material properties after exposure to complex wear mechanisms. The wear methods and their sequence were designed to mimic both mechanical and chemical impact during an anticipated daily oral hygiene and diet pattern in an individual. The observed deterioration of Filtek Z250^®^, Fuji IX GP^®^ and Equia Forte^®^ following the cumulative impact of wear might be representative of their respective material groups. Provided that Filtek Z250^®^ is a typical representative of CR materials, the observed highest final hardness value (Table 2) and the smallest substance loss (Figure 4) compared to the GI and GH materials, indicate that it clinically may be the preferable material for posterior restorations being exposed to the largest chewing forces. Despite similar hardness and roughness values of the GI and GH, the substance loss of Equia Forte^®^ was most likely smaller than that of Fuji IX^®^, as the latter could not be measured by optical profilometry (Figure 4).

Mechanical properties such as surface roughness and hardness are characteristics of the materials’ durability in the oral environment [28,29]. Moreover, increased surface roughness may contribute to facilitated microbial colonization and accumulation of biofilm potentially leading to inflammation of the soft oral tissues [30]. To agree on a critical roughness value for a certain material would not be feasible as such a value would be dependent on several factors such as the material composition, finishing and polishing procedures, as well as measuring technique and instruments [31,32]. According to Bollen et al. [33], the critical surface roughness (Ra) for microbial colonization of a restorative dental material is 0.2 µm, and higher values are related to increased plaque maturation. Following wear exposure in the current study, the surface roughness (Ra) value was higher than 0.2 µm for all material groups, indicating higher plaque susceptibility. The several-fold increase in roughness of Filtek Z250^®^ following wear exposure could be visualized by marks from the toothbrush bristles, which was not seen in specimens of the two other materials. The profilometer instrument capacity was apparently sufficient to detect the highest increase in surface roughness in Filtek Z250^®^. However, the fact that the wear caused material flakes to come off the Fuji IX GP^®^ and Equia Forte^®^ specimens, disabled the instrument to measure realistic roughness changes. We speculate that without the flaking off of the latter two materials, the roughness values would have increased for these materials (Table 2).

The standardized brushing procedure used in the current study to mimic 6 months of tooth brushing, represented an exaggerated abrasion treatment. The deterioration observed in this study may have been more pronounced than under clinical conditions. Clinically, saliva and biofilm are protective factors against wear. Hardness is a characteristic of a restorative material that may influence polishing capacity, scratching occurrence and the resistance to load application and plastic deformation [34,35]. Usually, it is validated by quantifying the depth or the surface area of an indentation created by an instrument within a certain time and with a defined force. Similar to roughness, hardness values might be influenced by the measuring procedures. According to the manufacturer, application of Equia Forte Coat^®^ strengthens the glass ionomer Equia Forte^®^, and thereby makes it more resistant to wear [36]. In the present study, neither the strengthening effect of a coat layer compared to effects on Equia Forte^®^ alone, nor the resistance to only one type of wear were studied.

The fact that the substance loss of Fuji IX^®^, in contrast to the other two materials, could not be analyzed due to the seemingly large depth and steepness of the groove indicates that this material had the largest loss of substance after the wear tests. Clinically, substance loss caused by wear is directly correlated with the ability of a restoration to withstand the grinding forces of the food and the antagonist teeth. Since no data for Fuji IX^®^ were obtained, further measurements of the substance loss of all three materials should be performed, including quantification, and comparing by making an impression of the worn area and a replica from the impression [35].

In general, chewing simulators and methods designed to test substance loss of restorative dental materials are based on different operational and methodological concepts, which makes it difficult to compare the results [37]. In human subjects, the magnitude of the initial biting force is approximately 20 N with an increase to 70 N on average at the end of the chewing cycle [38]. The average length of one chewing cycle is about 0.8 − 1 s with the sliding distance less than 1 mm [39]. These parameters were set for the chewing device constructed for the purpose of this study in an attempt to standardize the procedure and to correlate to the *in vivo* wear. Antagonists, the epoxy-embedded stainless steel ball used in the current investigation, are commonly used in the wear challenges for both localized and generalized wear simulation studies [40]. A disadvantage of using a metal antagonist is that the clinical relevance is inferior to an e.g. ceramic antagonist. However, applying a ceramic material antagonist would introduce another restorative material. Although enamel would be the best antagonist, for practical reasons (variation in antagonist shape, time required to mechanically produce a unique antagonist), it is less suitable for use in wear tests in comparison to other materials such as stainless steel or ceramics [41]. A stainless steel antagonist has a similar or slightly lower hardness value in comparison to tooth enamel [42]. Therefore, it was expected that plastic deformation with material transference (adhesive wear) also took part in the wear process [43]. The antagonists showed no visually observed surface changes. The material wear particles may have acted as an abrasive medium. The sliding distance was found to be twice the diameter of the antagonist diameter to enable material particles to be washed away, and thereby to reduce the abrasive effect [44]. Since in this attrition test, the sliding movement was 8 mm and the diameter of the antagonist was 4 mm, the effect of abrading particles could be considered as minor.

Several studies have reported the negative effect of different acidic drinks to restorative materials, such as change in surface roughness and color [3,44–48]. In this study, the sequence of the conducted wear tests might have influenced the postexperimental shades, since the specimens were exposed to the brushing procedure prior to the acidic challenge. Exposure of dental materials to a combination of brushing and immersion in an acidic drink was shown to result in more pronounced color change than when the materials were exposed to the acidic drink alone [48]. Therefore, continuous brushing and exposure to the abrasive slurry while the specimens were deprived of saliva might have rendered the materials prone to shade changes.

Despite the carefully controlled parameters of the exposures and characterization parameters in the present study, limitations were, for example, mimicking mandibular movement, chewing forces and saliva presence [27]. These are factors that are not entirely replicable. Furthermore, spectrophotometric color measurements would have improved detection of appearance changes. We acknowledge the clinically significant inter-individual variation in tooth brushing technique, food intake habits and chewing cycle length depending on the individuals’ preferences, age, gender or presence of oral and systemic diseases [49–51]. Such variation in factors are not encompassed in *in vitro* studies. Therefore, in addition to the reports from the laboratory studies, clinical, controlled studies aiming to understand the tooth wear mechanisms in the human oral environment, and their effect on restorative dental materials are warranted.

## Conclusion

The changes in mechanical properties following sequential wear exposure, mimicking abrasion, erosion and attrition, indicate that the composite resin, represented by Filtek Z250^®^, was more resistant than the glass ionomer, Fuji IX GP^®^, and the glass hybrid, Equia Forte^®^.
